# Facteurs associés à une issue défavorable chez les brûlés hospitalisés

**DOI:** 10.5588/pha.23.0007

**Published:** 2023-08-01

**Authors:** F. Niyonzima, H. Mboma Kamosi, J. Soro, O. Ntihabose, D. Hehadji, E. Briskin

**Affiliations:** 1 Médecins Sans Frontières (MSF), Bujumbura, Burundi; 2 Ministère de la Santé publique et de la Lutte contre le Sida, Bujumbura, Burundi; 3 MSF, Conakry, Guinée; 4 MSF Luxembourg Operational Research Unit, Luxembourg, Luxembourg

**Keywords:** mortalité, traumatisme, ressources limitées

## Abstract

**CONTEXTE:**

En 2015, Médecins Sans Frontières a ouvert un Centre de Traumatologie Arche Kigobe à Bujumbura, Burundi, pour prise en charge des victimes des violences, et a élargi en 2016 les critères d’admission aux brûlures, sans unité spécialisé pour leur prise en charge.

**OBJECTIF:**

Etudier les facteurs associés à une issue défavorable (décès, référés, et sortis contre avis médical), chez les brûlés hospitalisés dans ce centre.

**MÉTHODE:**

Ceci est étude descriptive et analytique rétrospective des patients brûlés hospitalisés

**RÉSULTATS:**

De 2016 à 2020, 477 patients ont été hospitalisés au Centre pour brûlure, dont 301 (63%) avaient moins de 5 ans, 169 (35%) étaient de sexe féminin, et 48 (10%) avaient une issue défavorable. L’anémie (OR 11 ; IC 95% 2,7–48), l’infection (OR 11 ; IC 95% 5,7–22), et l’inhalation de fumée (OR 28 ; IC 95% 7–111), étaient parmi les principaux facteurs associés à une issue défavorable.

**CONCLUSION:**

Même dans les contextes à ressources limitées, pour minimiser les issues défavorables liés à l’inhalation et l’infection chez les brûlés, un circuit d’isolement septique, la formation, un service de bactériologie, et les appareils de ventilation en pression positive continue pourraient être mis en place.

## Abstract (en)

**BACKGROUND:**

In 2015, Médecins Sans Frontières opened the Arche Kigobe Trauma Centre in Bujumbura, Burundi, to treat victims of violence, and in 2016 extended the admission criteria to burns, without a specialised unit to treat these.

**OBJECTIVE:**

To study the factors associated with an unfavourable outcome (death, referrals and discharges against medical advice) in burn patients hospitalised at this centre.

**METHOD:**

This is a retrospective descriptive and analytical study of hospitalised burn patients.

**RESULTS:**

From 2016 to 2020, 477 patients were hospitalised at the Centre for burns, of whom 301 (63%) were less than 5 years old, 169 (35%) were female, and 48 (10%) had an unfavourable outcome. Anaemia (OR 11, 95% CI 2.7–48), infection (OR 11, 95% CI 5.7–22), and smoke inhalation (OR 28, 95% CI 7–111) were among the main factors associated with an unfavourable outcome.

**CONCLUSION:**

To minimise adverse outcomes related to inhalation and infection in burn patients, a septic isolation circuit, training, a bacteriology service and continuous positive airway pressure could be implemented even in resource-limited settings.

Les brûlures sont en cause de 180 000 décès chaque année, selon l’OMS. Près des deux tiers des cas sont enregistrés dans les régions d’Afrique et d’Asie du Sud-Est.^1^

L’évolution des cas de décès dans le temps est variable selon les conditions socio-économiques des pays. Si une baisse de la létalité chez les brûlés a été notée dans les pays à revenus élevés grâce à une amélioration des conditions de prise en charge, le plateau technique est limité, comme l’infrastructure non adaptée, limite en matériel et équipement, formation insuffisante, et absence de service de bactériologie performant, dans les pays à revenus faible ou intermédiaire (PRFI). Dans une étude réalisée au Cameroun, entre 2008 et 2015, 440 cas de brûlés ont été enregistrés, parmi lesquels 23,4% étaient décédés. Dans cette étude, les causes de brûlures les plus courantes étaient les flammes, l’électricité et l’eau chaude.^1^

Les décès chez les patients brûlés font suite à de nombreuses complications (infections graves, choc hypovolémique, insuffisance rénale, rhabdomyolyse, troubles chimiques, syndrome compartimental) qui peuvent être favorisées par les consultations tardives, certaines pratiques comme l’application de produits traditionnels ou encore le recouvrement des plaies par du matériel souillé.^2^

Au Burundi, selon les données officielles publiées en 2004, les brûlures étaient parmi les cinq causes majeures de morbidité et de mortalité dans le pays.^3^ En 2019, en moyenne 272 cas de décès par brûlures ont été officiellement notifiés.^4^ En 2017, selon l’annuaire des statistiques sanitaires, avec 4 143 cas, les brûlures représentaient la deuxième cause justifiant des interventions ortho-traumatologiques dans le pays.^3^ Le Centre de Traumatologie Arche Kigobe figurait parmi les trois structures de santé ayant rapporté le grand nombre d’interventions sur les brûlés.^3^ Des cas de décès ont été enregistrés chez les brûlés hospitalisés au Centre de Traumatologie Arche Kigobe.

Cette étude avait pour objectif général d’étudier les facteurs associés à une issue défavorable, y compris la mortalité, chez les brûlés hospitalisés au Centre de Traumatologie Arche Kigobe, au Burundi, du 1^er^ février 2016 au 29 février 2020 ; et comme objectifs spécifiques de décrire les caractéristiques démographiques et les profils cliniques des patients, décrire le devenir des patients admis et d’identifier les facteurs associés à une issue défavorable.

## MÉTHODOLOGIE

Il s’agissait d’une étude descriptive et analytique basée sur les données rétrospectives des patients brûlés hospitalisés au Centre de Traumatologie Arche Kigobe du 1^er^ février 2016 au 29 février 2020.

### Contexte général

Le Burundi est un pays situé à cheval entre l’Afrique de l’Est et l’Afrique centrale. La population du Burundi est estimée à 12,26 millions d’habitants en 2021. À la suite de la crise électorale de 2015, le Burundi a été confronté à une instabilité politique accompagnée des violences.^5^

La prise en charge des brûlures en hospitalisation se fait partout dans le pays au niveau des hôpitaux de référence nationale, hôpitaux régionaux et de district. Cependant les ressources, connaissances, et plateaux techniques pour la prise en charge de brûlures graves restent limités (Tableau 1).

**TABLEAU 1:** Plateau technique pour la prise en charge des patients brûlés hospitalisés à l’Arche Kigobe

Disponible	Recommandé mais non disponible
ICU de niveau 1 avec 4 chambres individuelles pas suffisamment ventilées, dont 2 avec possibilité de monitorage multiparamétrique pour patients critiques et suite des soins en salle d’hospitalisation pour les patients améliorés.	Sas : petit vestibule avant l’ICU pour éviter une communication directe
Concentrateurs d’oxygènes de 5 et 10 L	Service de bactériologie-microbiologie
Une salle d’opération utilisée aussi pour la chirurgie digestive	Carbapénèmes (méropénem), avec prescription sur avis d’un Infectiologue et/ou selon les résultats de l’antibiogramme
Une salle d’opération pour les brûlés utilisée aussi pour la chirurgie digestive	ICU équipé de respirateurs
Possibilité d’isolement contact	Circuit pour isolement septique
	Chirurgie reconstructrice

ICU = intensive care unit.

### Contexte spécifique

En juin 2015, MSF a ouvert un Centre de Traumatologie Arche Kigobe à Bujumbura en vue d’appuyer le Ministère de la Santé Publique et de la Lutte contre le Sida dans la prise en charge des cas de traumatismes issus des violences liées à la crise électorale du moment. MSF offre les soins gratuitement.

Le Centre a par la suite élargi ses critères d’admission aux victimes des accidents de la voie publique, domestiques, professionnels et des brûlures. Les brûlés sont finalement retirés des critères d’admission du Centre à partir de mars 2020.

### Population de l’étude

La population étudiée était constituée par tous les patients brûlés admis en hospitalisation au Centre de Traumatologie Arche Kigobe du 1^er^ février 2016 au 29 février 2020. Les patients référés ailleurs après admission l’avaient été pour limitation du plateau technique du Centre et d’autres sont sortis contre avis médical. En raison d’un pronostic vital non maîtrisé au moment de la sortie de ces patients et la non-disponibilité de données sur leur issue finale, ces patients ont été considérés comme à issue défavorable de la même façon que les décédés. Les patients sortis guéris ont été considérés comme à issue favorable.

### Collecte des données

Les données pour cette étude ont été extraites de la base de données de suivi de routine des patients admis en hospitalisation complétée avec les données des outils primaires de collecte (registres et dossiers patients).

La base de données a subi un contrôle de qualité pour vérifier leur complétude, leur cohérence et leur exactitude pour cette étude.

Les variables utiles à l’étude : caractéristiques démographiques, caractéristiques cliniques, causes des brûlures, complications ainsi que l’issue des patients ont été collectées. Les patients avec un numération de formules sanguines et protéine C-réactive positive pour l’infection étaient considérés d’avoir une infection confirmée.

### Analyse des données

Les données étaient analysées dans Epi Info v7.2.5.0 (Centers for Disease Control and Prevention, Atlanta, GA, Etats Unis) et R v4.1.3 (R Computing, Vienne, Autriche). Les statistiques descriptives, y compris les moyennes, les médianes, et les taux, selon le type de variable, ont été calculées pour chaque variable. Pour déterminer les associations entre les variables catégorielles et l’issue défavorable, le test du χ^2^, le Fisher exact test, et les *odds ratios* (OR) ont été utilisés. Le test de Mann–Whitney a été utilisé pour déterminer les associations entre les variables continues avec distribution non-normal et l’issue défavorable, et les OR ont été calculés. Pour tous les tests statistiques, le niveau de signification était fixé à 0,05 (intervalle de confiance à 95% [IC 95%]).

### Considérations éthiques

L’approbation du Comité National d’Ethique du Burundi, Bujumbara, Burundi, a été donnée le 02 septembre 2022 sous la décision CNE/26/2022 et le Comité d’éthique de Médecins Sans Frontières MSF Ethics Review Board, Génève, Suisse, a marqué son exemption à l’étude via l’autorisation du Directeur Médical du Centre Opérationnel Bruxelles, Belgique.

## RÉSULTATS

De février 2016 à février 2020, 477 patients ont été hospitalisés à l’Arche Kigobe pour brûlure, dont 429 (90%) avec issue favorable et 48 (10%) avec issue défavorable (Figure 1).

**FIGURE:**
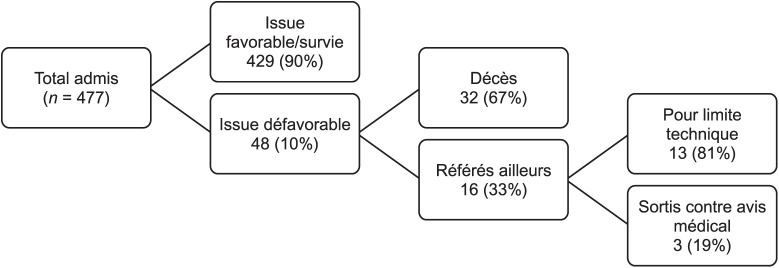
Description des patients admis en hospitalisation pour brûlure au Centre de ­Traumatologie Arche, au Burundi, de février 2016 à février 2020.

Parmi ces patients, 301 (63%) avaient moins de 5 ans avec l’âge médian de 3 ans (intervalle interquartile 2–10) ; et 169 (35%) des patients étaient de sexe féminin (Tableau 2). Plusieurs facteurs étaient associés à une issue défavorable. Les patients anémiques (la seule pathologie sous-jacente avec association significative) avaient 11 fois (IC 95% 2,7–48) la probabilité d’avoir une issue défavorable que ceux sans pathologie sous-jacente. Les patients avec un délai d’arrivé de plus de 24 h était moins probable d’avoir une issue défavorable que les patients qui se présentaient dans un délai de moins d’une heure, avec un OR de 0,3 (IC 95% 0,12–0,9). L’inhalation de fumée était fortement associée à l’issue défavorable, avec un OR de 28 (IC 95% 7–111). Un accroissement dans la surface cutanée brûlée, et une profondeur de brûlure de deuxième ou troisième dégrée, étaient associés à une issue défavorable. La présence de toutes les complications examinées, sauf syndrome compartimentale et insuffisance rénale, était significativement liée à une issue défavorable. Chaque jour de plus d’hospitalisation a été associé à une probabilité plus élevée d’avoir une issue défavorable.

**TABLEAU 2:** Caractéristiques démographiques, profils cliniques et facteurs associés à l’issue des patients brûlés hospitalisé au Centre de Traumatologie Arche Kigobe, février 2016–février 2020

	Total (*n* = 477) *n* (%)	Issue défavorable (*n* = 48) *n* (%)	Issue favorable (*n* = 429) *n* (%)	OR (IC 95%)	*P*-value (χ^2^-ou Fisher Exact)
Facteurs démographiques					
Catégorie d’âge, ans					
0–5	301 (63)	27 (56)	274 (64)	0,73 (0,40–1,33)	0,3
>5	176 (37)	21 (44)	155 (36)	Référence	
Sexe : femme	169 (35)	21 (44)	148 (35)	1,48 (0,81–2,70)	0,2
Facteurs cliniques					
Pathologies sous-jacentes					<0,01
Malnutrition	3 (0,6)	1 (2,1)	2 (0,5)	5,7 (0,5–65)	0,2
Paludisme	13 (2,7)	3 (6,3)	10 (2,3)	3,4 (0,9–13)	0,1
Epilepsie	17 (3,6)	3 (6,3)	14 (3,3)	2,5 (0,7–9,0)	0.2
Anémie	8 (1,7)	4 (8,3)	4 (0,9)	11 (2,7–48)	<0,01
Autre*	13 (2,8)	3 (6,3)	10 (2,3)	3,4 (0,9–13)	0,1
Aucune	423 (89)	34 (71)	349 (91)	Référence	
Agent causal de la brûlure					<0,01
Liquides chauds	391 (82)	28 (58)	363 (85)	Référence	
Flammes	58 (12,2)	18 (38)	40 (9,3)	5,8 (3,0–11)	<0,01
Produit chimique	3 (0,6)	1 (2,1)	2 (0,5)	6,5 (0,6–73)	0,21
Courant électrique	18 (3,8)	1 (2,1)	17 (4,0)	0,76 (0,1–6)	<0,01
Contact avec objet chaud	6 (1,3)	0 (0,0)	6 (1,4)	—	1
Données manquantes	1 (0,2)	0 (0,0)	1 (0,2)	—	1
Localisation de la brûlure					<0,01
Visage	15 (3,1)	0 (0,0)	15 (3,5)	0,0 (non-défini)	0,1
Crâne	1 (0,2)	0 (0,0)	1 (0,2)	0,0 (non-défini)	1
Cou	1 (0,2)	0 (0,0)	1 (0,2)	0,0 (non-défini)	1
Tronc	11 (2,3)	1 (2,1)	10 (2,3)	0,53 (0,07–4,3)	1
Fesses	7 (1,5)	0 (0,0)	7 (1,6)	0,0 (non-défini)	0,6
Membres supérieurs	99 (21)	0 (0,0)	99 (23)	0,0 (non-défini)	<0,01
Membres inférieurs	52 (11)	1 (2,1)	51 (12)	0,1 (0,01–0,77)	<0,01
Localisation multiple	291 (61)	46 (96)	245 (57)	Référence	
Lésion par inhalation	11 (2,3)	8 (17)	3 (0,7)	28 (7–111)	<0,01
Profondeur					<0,01
1^er^ degré	12 (2,5)	0 (0,0)	12 (2,8)	Référence	
2^ème^ degré	435 (91)	36 (75)	399 (93)	—	0,6
3^ème^ degré	30 (6,3)	12 (25)	18 (4,2)	—	<0,01
Pourcentage de la surface cutanée brûlée^†^	11 (10–12)	33 (26–40)	9 (8,4–9,6)	1,1 (1,1–1,2)	<0,01
Présence d’une lésion majeure associée	7 (1,5)	0 (0,0)	7 (1,6)	0,0 (non-défini)	1
Application de produit avant l’arrivée	11 (2,3)	1 (2,1)	10 (2,3)	0,9 (0,11–7,12)	0,91
Délai d’arrivée					0,07
<1 h	143 (30)	20 (42)	123 (29)	Référence	
1 h–<6 h	123 (26)	15 (31)	108 (25)	0,9 (0,4–1,8)	0,7
6 h–<24 h	110 (23)	8 (17)	102 (24)	0,5 (0,2–1,1)	0,1
≥24 h	101 (21)	5 (10)	96 (22)	0,3 (0,12–0,9)	0,02
Complications					
Hypothermie	7 (1,5)	13 (27)	0 (0,0)	—	<0,01
Déshydratation	4 (0,8)	3 (6,3)	1 (0,2)	29 (3–280)	<0,01
Choc septique	13 (2,7)	13 (27)	0 (0,0)	—	<0,01
Infection confirmée	52 (11)	22 (46)	30 (7)	11 (5,7–22)	<0,01
Rhabdomyolyse	2 (0,4)	2 (4,2)	0 (0,0)	—	<0,01
Insuffisance rénale aiguë	1 (0,2)	1 (2,1)	0 (0,0)	—	0,1
Syndrome compartimental	3 (0,6)	0 (0,0)	3 (0,7)	0,0 (non-défini)	1
Fièvre	185 (39)	32 (67)	153 (36)	3,6 (1,9–6,8)	<0,01
Jours d’hospitalisation^†^	15 (13–17)	8,4 (3,8–12)	16 (13–17)	9,3 (8,8–9,7)	<0,01

*Otite moyenne suppurée, affection digestive chronique, affection virale chronique, affection pulmonaire chronique, dermatose et psychopathie.

^†^Résultats exprimés en moyenne et IC à 95%. *P*-value du test Mann-Whitney.

OR = odds ratio ; IC = intervalle de confiance.

## DISCUSSION

Il s’agit de la première étude menée au Burundi pour investiguer les facteurs associés à une issue défavorable des patients hospitalisés pour brûlure, réalisée au Centre de Traumatologie Arche Kigobe. La population de l’étude était majoritairement (63%) des enfants de moins de cinq ans. Dix pourcent des patients ont eu une issue défavorable, ce qui est similaire aux taux de mortalités de 12% et 17% trouvés dans les contextes similaires en Ethiopie et au Kenya et inférieur au taux de mortalité de 41% trouvé au Cameroun.^6–9^

Un pourcentage de surface cutanée brûlée élevé, une brûlure causée par les flammes, une longue durée de séjour à l’hôpital, et une profondeur de deuxième ou troisième degré sont typiquement associés à une issue défavorable chez les patients brûlés, et ce même effet a été démontré ici.^10–13^ Un court délai d’arrivé à l’hôpital a également été démontré comme un facteur associé à l’issue défavorable ici et dans d’autres études, probablement en lien avec l’urgence de besoin de soins en rapport avec la gravité des lésions de brûlure.^14^

Les patients anémiques avaient 11 fois la probabilité d’avoir une issue défavorable que ceux sans pathologie sous-jacente, une association qui a été trouvée dans plusieurs études sur les patients hospitalisés avec brûlure, étant donné que l’anémie peut retarder la guérison ; ceci a aussi été associé au développement des infections chez les patients brûlés.^15,16^ La malnutrition a été trouvée comme un facteur associé à l’issu défavorable dans plusieurs autres études des patients brûlés.^17,18^ Cette étude a trouvé la même tendance, mais l’effet n’était pas significatif, probablement en lien avec les sous-effectifs des participants identifiés avec malnutrition car n’étant pas systématiquement dépistée.

L’inhalation de fumée ne crée pas les lésions qui sont visibles sur l’extérieur du corps du patient, mais les effets à l’intérieur des poumons sont connus d’être dangereux.^19^ Ce facteur était fortement associé à l’issue défavorable, avec un OR de 28 (IC 95% 7–111). Dans un centre de traitement de brûlés régional en Angleterre, le *hazard ratio* associé à l’inhalation de fumée était de 2.14 (IC 95% 1.12–4.09).^20^ L’intubation avec un respirateur, ou à défaut, l’usage d’un appareil de ventilation en pression positive continue (PPC) est recommandé pour les patients avec inhalation de fumée,^21,22^ mais seulement les concentrateurs d’oxygène étaient disponibles dans le Centre de Traumatologie Arche Kigobe. Cette faillite de plateau technique pourrait avoir contribué à la mortalité élevée observée chez ces patients.

L’infection et les signes y associés comme la fièvre et le choc septique, sont connus d’être associés à l’issue défavorable des patients brûlés, une association qui a été trouvée ici et dans d’autres pays à moyennes et faibles ressources.^10,23^ Pour prévenir les infections chez les patients brûlées, un centre de traitement pour les brûlées est recommandé d’intégrer certains standards, y compris un système de traitement et de contrôle de l’air réduisant le risque de contamination microbienne par voie aérienne, et d’avoir un service de bactériologie afin d’ajuster l’antibiothérapie, deux choses qui étaient notamment absentes à l’Arche Kigobe.^24^

Comme limites, les patients avec conditions médicales nécessitant une prise en charge non disponible à l’Arche Kigobe et les patients sortis contre avis médical sont partis poursuivre les soins ailleurs. L’absence de données sur les structures de destination finale ainsi que leurs issues ont constitué une limite à notre étude. Vu que la plupart de ces patients avaient un mauvais pronostic vital, et que d’autres centres avec un plateau technique beaucoup plus élevé ne sont pas disponibles dans le pays, ces patients ont été considérés comme à issue défavorable de la même façon que les décès pour minimiser les biais d’interprétation.

L’application ou non des produits avant l’arrivée à l’hôpital ainsi que les types de produits sont des variables susceptibles d’influencer l’issue des brûlés, mais ces informations n’étaient pas systématiquement mentionnées dans les dossiers. Une place réservée à la récolte de cette information pourra être ajoutée sur la fiche d’admission du patient brûlé et remplie systématiquement.

Une force de cette étude est qu’elle est la première à décrire les facteurs associés à l’issu défavorable des patients hospitalisés avec brûlures au Burundi, et elle répond à une faillite connue dans la littérature.^6^

En conclusion, même si la construction d’un centre spécialisé pour la prise en charge des brûlures est recommandée dans l’avenir, cette étude a mis en évidence d’autres interventions qui peuvent quand même être implémentées à défaut, dans les contextes à ressources limitées, y compris les contextes humanitaires. Le centre concerné dans cette étude n’avait pas d’infrastructure adaptée pour optimiser la prévention et le contrôle des infections chez des brûlés, et l’infection a été fortement associée à l’issue défavorable. Un circuit d’isolement septique, la formation, et un service de bactériologie, pourraient être mis en place pour minimiser les issues défavorables liées aux infections. De plus, à défaut des respirateurs, les PPCs pourraient être rendus disponibles pour améliorer l’issue des patients avec lésions par inhalation.

### Remerciements

Cette recherche a été menée dans le cadre de l’Initiative de recherche opérationnelle et de formation structurée (SORT-IT), un partenariat mondial dirigé par le Programme spécial de recherche et de formation sur les maladies tropicales de l'Organisation mondiale de la santé (OMS/TDR). Le modèle est basé sur un cours élaboré conjointement par l'Union internationale contre la tuberculose et les maladies respiratoires (L'Union) et Médecins Sans Frontières (MSF/Doctors Without Borders). Le programme spécifique, SORT-IT, qui a donné lieu à cette publication a été organisé par MSF spécifiquement pour la recherche en langue française.

Le programme a été financé par La Fondation Veuve Emile Metz-Tesch, Luxembourg. Le financeur n’a joué aucun rôle dans la conception de l’étude, la collecte et l’analyse des données, la décision de publier ou la préparation du manuscrit.
